# Training Australian Dietitians in Behavior Change Techniques Through Educational Workshops: Protocol for a Randomized Controlled Trial

**DOI:** 10.2196/49723

**Published:** 2023-12-04

**Authors:** Hayley Breare, Barbara Mullan, Deborah A Kerr, Chloe Maxwell-Smith

**Affiliations:** 1 School of Population Health Curtin University Bentley Australia; 2 Behavioural Science and Health Research Group Curtin University Bentley Australia

**Keywords:** BCT, behavior change, COM-B, continuing education, dietitians, health professional, intervention, professional development, psychology, randomized controlled trial, training, workshop

## Abstract

**Background:**

The use of education alone as a technique to change behavior in interventions is usually insufficient, particularly in health interventions. Behavior change techniques have been shown to be effective in fostering positive changes in health behaviors such as diet and physical activity. The upskilling of health professionals can increase perceived capability and motivation toward eliciting change in clients’ behaviors. However, to date, dietitians have received limited training in behavior change and have expressed a need for continuous professional development.

**Objective:**

The study objectives are to (1) develop and evaluate the effectiveness and acceptability of two 2-hour behavior change workshops on changing dietitians behavior (ie, range of behavior change techniques used and frequency of use) across 3 time points; (2) determine if participation in these workshops will elicit changes in dietitians’ perceived capability, opportunity, and motivation toward using behavior change techniques; and (3) determine the acceptability of the training and its application in practice by dietitians.

**Methods:**

We will recruit registered dietitians (N=140) in Australia to participate in this randomized controlled trial. Participants will be randomly assigned to either the intervention or 3-month waitlist control condition and complete outcome measures at 3 time points: baseline, after the workshop, and follow-up at 3 months. Both groups will complete 2 workshops on behavior change that are guided by the COM-B (Capability, Opportunity, Motivation, and Behavior) Model and embedded with behavior change techniques. The primary outcome is changes in behavior, (ie, the range of behavior change techniques used and their frequency of use). Secondary outcomes include changes in perceived capability, opportunity, motivation, and preparedness as a health professional toward delivering behavior change techniques. The acceptability of the workshops will also be assessed after the workshop through the postworkshop survey and semistructured interviews. A series of 2-way repeated measures ANOVAs and regressions will be used. Qualitative data will be analyzed using thematic analysis.

**Results:**

Participant recruitment commenced in June 2023. The results of the study are expected to be published in November 2024. The results will allow us to assess comparisons between the intervention and waitlist control groups, as well as changes in perceived capability, opportunity, motivation, and preparedness over a 3-month period. It will also provide an understanding of the acceptability of the training as a form of continuous professional development for dietitians.

**Conclusions:**

If found to be effective, the results of this 2-arm randomized controlled trial will guide future training and continuous professional development for health professionals in changing behavior in practice. Our findings will contribute to our understanding of the application of behavior change techniques in practice with clients and identify components of COM-B where dietitians may need future support.

**Trial Registration:**

ACTRN12623000525684; https://www.anzctr.org.au/ACTRN12623000525684.aspx

**International Registered Report Identifier (IRRID):**

PRR1-10.2196/49723

## Introduction

Chronic diseases such as cardiovascular disease, chronic obstructive pulmonary disease, cancer, and diabetes are among the most common noncommunicable diseases, which contribute to the burden of disease in Australia [1]. Approximately 38% of this burden of disease could be prevented by addressing behavioral determinants of disease such as overweight (including obesity) and dietary risk behaviors [2]. The majority of Australians do not meet the Australian government dietary guidelines for fruit and vegetable consumption, with almost 50% of adults eating fewer than 2 serves of fruit and 92% not eating the recommended 5 serves of vegetables daily [3]. Therefore, there is a need for behavior change interventions and health promotion across primary health care settings. However, while health professionals typically focus on education for clients, consumers, or patients (from here forward, any patient, client, or consumer will be referred to as clients), this is usually insufficient for facilitating behavior change and leads to missed opportunities in practice [4]. Behavior change techniques, which are considered the “active ingredients” of intervention design, have been shown to be more effective in improving client outcomes compared to those without, particularly in dietary interventions [5,6]. As such, it is important that health professionals are equipped with the capability and motivation to effectively facilitate behavior change among clients.

Brief opportunistic interventions in routine consultations have been effective for eliciting change in clients’ behavior [7,8]. However, health care professionals report barriers to the implementation of behavior change techniques, such as poor confidence in individual skills and knowledge of behavior change, time constraints, priorities, and access to sufficient training [4]. Dietitians engaging in both in-person and telehealth consultations are in an optimal role where they can reach both metropolitan and rural clients to deliver health-behavior change interventions [9]. Additionally, many dietitians have expressed an unmet need for knowledge and skills in behavior change [10,11].

The understanding of behavior change theories and techniques can help health professionals understand behavior and the mechanisms that elicit or facilitate behavior change [12]. Theoretical models, such as the COM-B (Capability, Opportunity, Motivation, and Behavior) model [13] can provide a framework for identifying and explaining the factors enabling or hindering changes in behavior, which can guide intervention design for effective behavior change [14]. The COM-B model posits that an individual’s capability (ie, physical and psychological capability), opportunity (ie, social and physical opportunity), and motivation (ie, reflective and automatic) interact in a bidirectional way and can explain behavior [14]. As such, interventions informed by these constructs have been effective in eliciting changes in knowledge and behavior [15,16], alongside the use of behavior change techniques [17,18]. Behavior change techniques can be considered the individual units or “active ingredients” that target components of behavior that need to change [6]. These components or units that form interventions can help us identify the parts of the intervention that work or do not work, providing valuable information for operationalizing effective intervention designs. The use of behavior change techniques can be effective in both changing the behavior of clients and health professionals [19,20]. For example, behavior change techniques such as goal setting, action planning, and self-monitoring have elicited behavior change in physical activity and food-related behaviors [21-24].

Despite the capacity of health professionals to foster behavior change in clients using behavior change techniques, no such training is consistently delivered to dietitians, particularly in formalized education [25]. Currently, the use of behavior change techniques used in dietary practice and dietary-based interventions is based around goal setting, problem-solving, social support, self-monitoring, and education-based techniques (eg, instructions on behavior and credible sources), which are consistent throughout the literature [5,26,27]. Therefore, the primary aim of the study is to develop and evaluate the effectiveness and acceptability of two 2-hour behavior change workshops on changing dietitians’ behavior, that is, changes in the number and frequency of behavior change techniques dietitians use in practice. It is anticipated that the workshops will prompt dietitians to use a larger variety and frequency of using these techniques in practice between baseline, after the workshop, and 3-month follow-up. A secondary aim is to determine if attendance at these workshops will demonstrate an increase in dietitians’ capability, opportunity, and motivation toward using these techniques. In these workshops, the use of behavior change techniques will underlie workshop material to target dietitians’ barriers across capability (eg, persuasion around capability), opportunity (eg, identifying barriers and problem-solving), and motivation (eg, focus on past success). Therefore, we hypothesize that engagement in these workshops will predict dietitians’ capability, perceived opportunity, and motivation toward using behavior change techniques from baseline and after the workshop to 3-month follow-up. Lastly, we also aim to determine the acceptability of the training.

## Methods

### Study Design and Procedure

**Figure 1:**
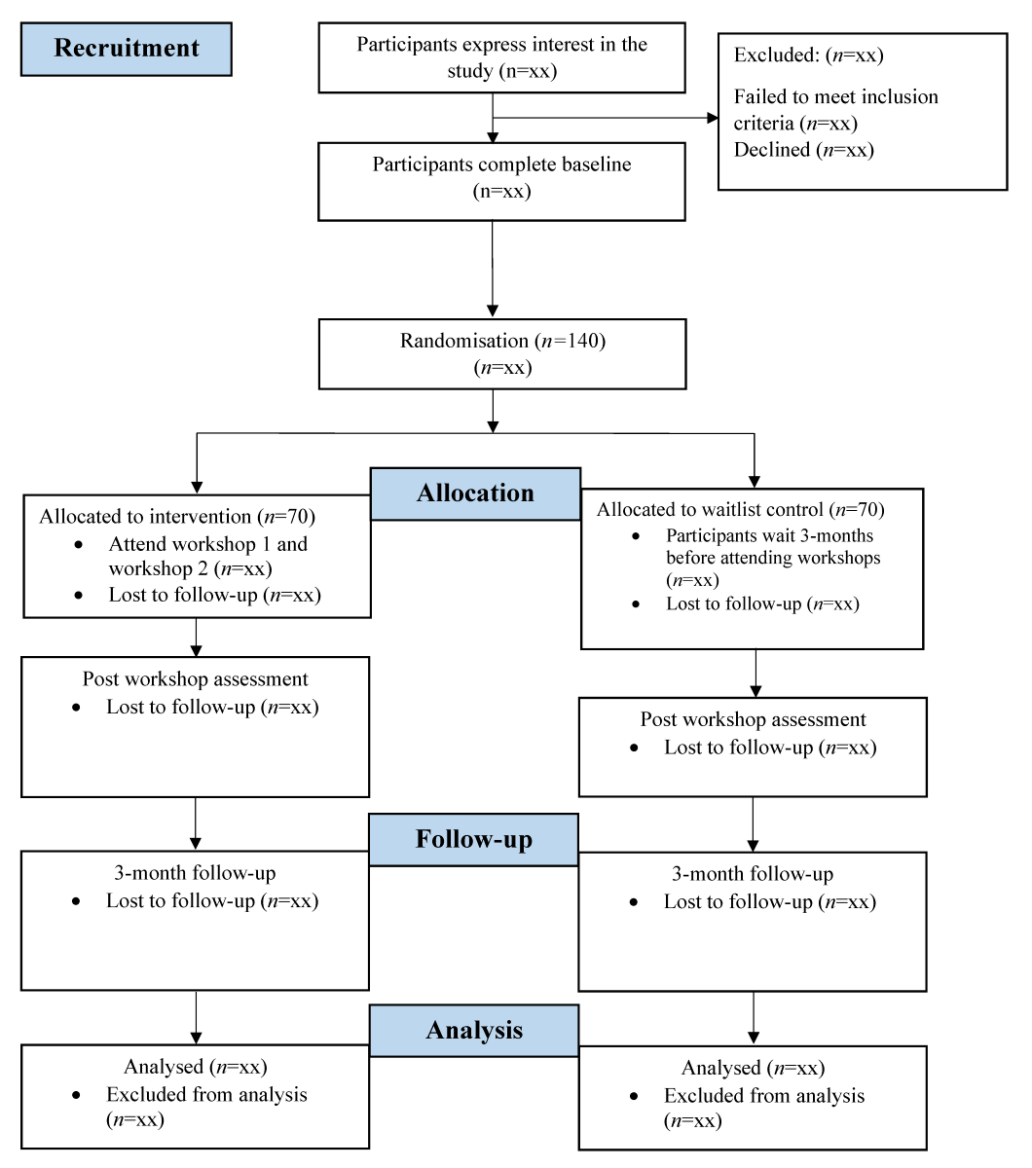
Outline of the trial.

This study is a 2-arm randomized controlled trial with a 3-month follow-up. Participants will complete a questionnaire at baseline, immediately after the intervention, and 3 months after the intervention. Participants will express their interest to participate in the study through a Qualtrics survey. Informed consent will be obtained at the start of this survey. Following this initial survey, dietitians will be randomized in blocks of 4 into either the intervention or a waitlist control condition (1:1). Block randomization will be conducted with allocation concealment from the active research team through the use of sealed, opaque envelopes. Emails will be sent to participants with the initial baseline questionnaire, and they will be informed if they can participate in the workshops immediately or if their available workshop will be in 3 months. Once dietitians have been randomized and received their baseline questionnaire, they have been enrolled in the study as participants. Both groups will participate in both workshops and complete a questionnaire immediately after participation in the second workshop and at the 3-month follow-up. The waitlist control group will be debriefed after the final questionnaire regarding their allocation to the waitlist control group. Postworkshop data collected from the waitlist control group will also be included in the final data set. Figure 1 shows a flowchart for each group’s progression in the trial. This protocol will adhere to the CONSORT (Consolidated Standards of Reporting Trials) and SPIRIT (Standard Protocol Items: Recommendations for Interventional Trials) guidelines for randomized controlled trials [28].

### Participants

Participants will be dietitians who are currently working in Australia and hold full membership with Dietitians Australia. Participants will be recruited using purposive sampling, through both social media advertisements and newsletters directed through nutrition and dietetic organizations, and through snowballing. Recruitment of participants will be staggered over a 12-month period. A similar intervention in behavior change for health professionals found medium to large effect sizes for changes in mechanisms of change (eg, perceived knowledge) [16]. Therefore, anticipating medium to large effect sizes, a-priori analysis was conducted in G power (Axel Buchner; *F*_1,82_=0.2; power =.80; α=.05) indicating that 42 participants in each group would be adequate [29]. This means a total sample size of 84. It is anticipated that there may be high attrition in this study. Therefore, we aim to recruit approximately 140 participants to ensure that if 50% of each group are lost to attrition, the proposed analysis will be sufficiently powered. This dropout rate of 50% is conservative given that similar interventions in training health professionals can report moderate (20%) to high (50%) attrition rates [20,30].

### Intervention Components

The workshops have been developed and piloted by a research team of psychology researchers and dietitians with clinical and teaching experience (Textbox 1). The main objectives for these workshops are intended for dietitians to be able to (1) understand the importance of behavior change, (2) define the characteristics of the COM-B model, and (3) identify opportunities to and implement behavior change techniques in practice. The first workshop will focus on developing knowledge of behavior change and the COM-B model [13]. The second workshop is focused on providing dietitians with behavior change techniques from the behavior change taxonomy [6]. These techniques were based on those shown to be effective in dietary interventions [31] and were chosen based on the most relevant techniques for practical use by dietitians. These workshops will be delivered both remotely through Teams (Microsoft Corporation) and in person. The design of these workshops is informed by behavior change techniques to target participants capability, perceived opportunity, and motivation to use behavior change techniques. These are delivered through activities and group discussions to evoke reflection, awareness of their own behavior, and problem-solving to overcome the barriers that exist across their own capability, perceived opportunity, and motivation (Multimedia Appendix 1 [6,19,32-37]).

**Outline of workshops.**

**Workshop 1**

- Understanding behavior
- Exploring behavior and behavior change
- Introduction to the Capability Opportunity Motivation-Behavior Model
- Case study
- Behavior change techniques
  What are behavior change techniques?
  Explore goal setting, problem solving, self-monitoring, and social support.
  Case study in small groups behavior change technique

**Workshop 2**

- Behavior change techniques
- Further explore 9 additional behavior change techniques and how they might apply in practice (eg, pros and cons and reattribution).
- Case study in small groups
- Relapse management and prevention

### Data Collection Methods

Once an expression of interest has been received through the Qualtrics survey, the participant will be asked to complete an initial baseline measure. In this baseline questionnaire, participants will be asked questions on their demographics (eg, age, gender, and highest level of qualification), current perceived knowledge and behavior (ie, use of behavior change techniques), the COM-B questionnaire [38], and preparedness as a health professional to deliver behavior change techniques. The same assessments (excluding demographics) will be repeated at the postworkshop and follow-up time points. At the postworkshop time point, the questionnaire will also include items on the acceptability of the workshops.

### Outcomes

The primary outcome of this study will be behavior change, which will be operationalized in 2 ways for dietetic practice. These outcomes include (1) a change in the number of behavior change techniques used and (2) a change in the frequency of behavior change techniques used. This change will be measured across the 3 time points. While this may not be an absolute measure of behavior change technique delivery, however, any change will capture the range of techniques taken from the workshops, which may be more indicative of the dietitians’ understanding and application of the COM-B model. To further capture the uniqueness of the behavior change techniques delivered by dietitians, the type of technique will also be measured across the 3 time points. Secondary outcomes include changes in perceived capability, opportunity, motivation, and preparedness as a health professional toward delivering behavior change techniques.

### Measures

Behavior will be measured with two items to determine (1) the use of behavior change techniques and (2) the frequency of use. The first item asks participants “Which of the following behavior change techniques do you use in practice?” Participants will indicate which of the 14 techniques they have used by rating their answer as either yes, no, or unsure. A total of 14 techniques were chosen based upon techniques used for dietary behavior change in the literature [31], as well as consultations with an advisory group of dietitians to determine the appropriateness and practicality of the techniques. A composite score will be calculated to determine the number of techniques used. A second item will also be included to identify how frequently dietitians use these techniques in practice, for example, “How often do you currently use behavior change techniques when interacting with clients?” Participants will rate this item on a 5-point Likert scale from never (0) to always (4).

Capability, opportunity, and motivation will be measured using the COM-B questionnaire [38], which has been adapted for delivering behavior change techniques when interacting with clients in health contexts. This measure includes 6 items and 6 domains that reflect each component of the COM-B model as outlined by Michie et al [13]. Participants will rate their level of agreement with each item on an 11-point scale, with items ranging from strongly disagree (0) to strongly agree (10). An example item includes “I have the PHYSICAL opportunity to deliver behavior change techniques when interacting with clients (eg, time, the necessary materials, reminders, and space).”

Perceived knowledge will be measured using 3 items related to knowledge of behavior change techniques. Each item will be rated on a 7-point slider scale. Participants will rate their level of understanding of behavior change techniques and their knowledge of 9 categories of techniques from “no understanding” (1) to “perfect understanding” (7). These categories are described in the behavior change taxonomy [39]. Scores for each statement will be summed to create a total perceived knowledge score. Item 3 will ask participants to rate their level of agreement with 3 statements relating to the use of behavior change techniques (ie, if they have used them before and if they know why they are useful). These statements are rated from “strongly disagree” (1) to “strongly agree” (7). Perceived knowledge will be assessed at the 3 time points.

The preparedness of dietitians to deliver behavior change techniques will be measured using a 6-item scale. This scale has been used with health professionals, such as pharmacists [40]. This measure is to determine if dietitians feel they are equipped to deliver these techniques as part of their consultations. Statements will be rated on a 5-point Likert scale from (1) strongly disagree to (5) strongly agree. An example includes “Dietitians need training in communication skills to enable them to deliver a behavior change techniques.”

### Process Evaluation

The process evaluation will assess dietitians’ acceptability of the 2 workshops. A total of 2 items will be included at the end of the postworkshop questionnaire, which was developed by Kothe and Mullan [41]. The first question will ask participants their level of agreeance on 14 keywords related to the facilitated workshops. For example, whether they felt the workshops were “needed, useful, appropriate, interesting, exciting, worthwhile, too short.” These will be scored on a 5-point Likert scale. The second question asks participants how satisfied they were with the workshops, rated on a 5-point Likert scale ranging from “very dissatisfied” (1) to “very satisfied” (5). At follow-up, participants will also be invited to complete a one-on-one exit interview through Microsoft Teams. These interviews will allow us to assess the experience and impact of the intervention (eg, barriers and enablers) and explore the acceptability and feasibility of the workshops. Interviews will be guided by the COM-B model [13] and are expected to take 30-45 minutes each. In addition, participants who choose to withdraw from the study will also be invited to complete 3 short open-ended questions regarding their reason for withdrawing from the study. This will provide insights into noncompleters experiences and attitudes toward the intervention.

### Statistical Analysis

#### Preliminary Analyses

Missing data will be managed during the data cleaning stage using case-wise deletion for missingness greater than 40%. Considerations for the use of multiple imputations as a method for addressing missing data will be made only if appropriate. Multiple imputation as a method for handling missing data is recommended for use in randomized controlled trials [42]. Assumption testing (eg, homogeneity of variances and normality) will also be conducted. If assumptions are not satisfied, appropriate transformations will be made. Dropout analyses using independent sample *t* tests (2-tailed) will identify any significant differences between participants who complete the study and those who drop out. Per protocol, intention-to-treat analyses will also be conducted as recommended for use in randomized controlled trials to reduce sample bias [43,44].

#### Primary and Secondary Outcomes

The primary and secondary outcome variables will be assessed at the baseline, postworkshop, and follow-up time points. A chi-square test will be used to assess if there are any differences between the intervention group and waitlist control at baseline. A 2-way repeated measures ANOVA will be conducted to compare the means between the intervention group and waitlist control group (between-subjects factor) over the 3 time points (within-subjects factor) for each of the primary and secondary variables. This analysis will be run to test for any time through conditional interactions. Demographics, such as age, gender, role of dietitian, and consultation delivery mode, will be included as covariates in the analysis. Multiple regression analyses will be used to determine whether changes (residual) in capability, opportunity, motivation, and health professional preparedness predict changes in behavior (range of use or frequency of use) at each of the 3 time points (while controlling for baseline behavior).

#### Qualitative Data

Interview data will be analyzed using thematic analysis to identify common themes concerning the impact of the intervention (eg, barriers or enablers experienced in practice) and to inform the acceptability and feasibility of the workshops [45].

### Ethical Considerations

Ethics approval was granted for this study by the Human Research Ethics Office on January 20, 2023 (HRE2023-0026). This project has also been approved by the Human Research Ethics Committee (HRE2023-0026) and the Australian and New Zealand Clinical Trials Registry (ACTRN12623000525684). Informed consent to participate in the study will be received during the initial recruitment stage before any data collection. Dietitians who are interested in participating will follow a Qualtrics survey link to the participant information sheet that details their involvement in the trial. Following this information, participants will be asked to consent to participate in the study, and if they choose to withdraw from the study, they will also consent to being contacted with follow-up questions. The workshops provided will be freely accessible and are able to be recorded as continuous professional development under Dietitians Australia.

### Research Data Plans

All research data collected as part of this project will be stored on the research drive of the university. Only the research team will have access to this data. Participant data will be collected and matched across the 3 time points based on their name, email address, and participant ID. All the data collected as part of this trial will remain confidential. After data collection, the 3 time points will be matched, and the data will be deidentified. Participants will be sent email reminders for each upcoming workshop and assessment.

## Results

Participant recruitment commenced in June 2023. Recruitment will be staggered over 12 months. Data analysis will commence in 2024, with the anticipated publication of results in 2024.

## Discussion

### Principal Findings

This trial will examine the effectiveness and acceptability of two 2-hour behavior change workshops and, to our knowledge, is the first training workshop to promote behavior change techniques among dietitians. There is evidence to suggest that training health professionals is beneficial in improving capability and motivation for using behavior change techniques in practice [20,33]. It is important that health professionals are confident in eliciting and identifying opportunities to facilitate brief opportunistic interventions with the goal of developing long-term behavioral maintenance. The findings of this study will provide insights into effective strategies for training of allied health professionals, such as dietitians, which could inform efforts to incorporate behavior change education into either formalized training or continuous professional development.

### Strengths and Limitations

A strength of this study is the use of a rigorous, co-designed process involving both experienced researchers in behavior change and a supervisory team of dietitians. Dietitians have been involved throughout the workshop design process and provided feedback to ensure terminology is consistent and information is tailored to nutrition and dietetic practice. Co-design provides valuable insights into stakeholders and consumer perspectives, allowing for a more targeted approach to intervention design [46]. A potential limitation that may arise during the study is that participants who do express interest are likely to have high motivation and potential knowledge of behavior change before the workshops. This may lead to only minor or no changes in capability, opportunity, and motivation, even if the workshops are effective, and may limit the generalizability of conclusions concerning uptake and interest in training.

### Conclusions

The examination of the effectiveness and acceptability of this trial will inform strategies for continuous professional development in behavior change. If found to be effective and acceptable by dietitians, there is potential for future training and professional development programs across allied health professionals who are in opportunistic and client-facing roles to target chronic disease prevention and management [47,48]. Our findings will also contribute to our understanding of translating skills around behavior change techniques into practice with clients and identifying those components of COM where dietitians may need future support.

## Appendix

Multimedia Appendix 1 Behavior change techniques mapped to workshop material.

## Abbreviations

COM-B: Capability, Opportunity, Motivation, and Behavior
CONSORT: Consolidated Standards of Reporting Trials
SPIRIT: Standard Protocol Items: Recommendations for Interventional Trials

## Data Availability

The data sets generated during and analyzed during this study are not publicly available due to privacy and ethical restrictions but are available from the corresponding author on reasonable request.
