# Supplementary material for: Training Australian Dietitians in Behavior Change Techniques Through Educational Workshops: Protocol for a Randomized Controlled Trial
Source: JMIR Res Protoc. 2023 Dec 4;12:e49723. doi: 10.2196/49723 (PMC10728788; doi:10.2196/49723)
Supplement: Multimedia Appendix 1 [file resprot_v12i1e49723_app1.docx]

**Appendix I. Behavior change techniques mapped to workshop material**

| **Application of BCT in workshop material** | **BCT applied**  **Labels from [1]** | **Capability** | **Opportunity** | **Motivation** |
| --- | --- | --- | --- | --- |
| Instructions on how to use behavior change techniques /application of the COM-B model | 4.1 Instruction on how to perform the behavior | Psychological capability |  |  |
| Participants will work through a case study in pairs/small groups to apply their knowledge of the COM-B model and behavior change techniques. | - 1. Problem solving   2. Action planning   3. Social support | Psychological capability | Social opportunity |  |
| Showing participants an image of a consultation and asking them to outline some of the barriers they might experience and then problem solve how they might overcome these barriers in practice. | - 1. Problem Solving   1.4 Action planning |  | Physical Opportunity |  |
| In workshop two, participants will have an opportunity to discuss any challenges they experienced in practice in implementing learning from the previous workshop. | 1.2 Problem solving  1.4 Action planning |  | Physical Opportunity |  |
| Asking participants to describe a time where they have used one of the behavior change techniques outlined and describe when it worked well with a that client. | 15.3 Focus on past success  1.6 Discrepancy between current behavior & goal |  |  | Reflective Motivation |
| Prompt participants to develop some strategies of what they can do to enable them to use these techniques in practice and create an action plan of how they will achieve this.  This might include setting goals, creating if-then plans, using cues that can be used over the next 3 months. | 8.3 Habit formation  1.1 Goal setting  1.4 Action planning  7.1 Prompts/cues |  |  | Automatic Motivation |
| At the end of workshop one, participants will be encouraged to create two goals related to the material delivered, prior to workshop 2. This might be shared with another participant/group. | 1.1 Goal setting  1.4 Action planning  1.9 Commitment  2.3 Self-monitoring (within their plan) |  |  | Automatic motivation |
| ***Note.*** *The behavior change techniques outlined have been embedded or recommended for use in interventions to upskill health professionals through continuous professional development or general training [6,19,32-37].* | | | | |
